# Structural and Charge Transport Properties of Composites of Phosphate-Silicate Protonic Glass with Uranyl Hydroxy-Phosphate and Hydroxy-Arsenate Obtained by Mechano-Chemical Synthesis Undergoing Hydration Changes

**DOI:** 10.3390/ma16010267

**Published:** 2022-12-27

**Authors:** Karolina Majewska, Maja Mroczkowska-Szerszeń, Rafał Letmanowski, Piotr Ryś, Wojciech Pudełko, Magdalena Dudek, Aldona Zalewska, Norbert Obarski, Lidia Dudek, Michał Piszcz, Grażyna Zofia Żukowska, Maciej Siekierski

**Affiliations:** 1 Inorganic Chemistry and Solid State Technology Division, Faculty of Chemistry, Warsaw University of Technology, ul. Noakowskiego 3, 00-664 Warsaw, Poland; 2 Oil and Gas Institute—National Research Institute, ul. Lubicz 25a, 30-350 Cracow, Poland; 3 Paul Scherrer Institut (PSI), Forschungsstrasse 111, 5232 Villigen, Switzerland; 4 Faculty of Fuels and Energy, AGH—University of Science and Technology, al. Mickiewicza 30, 30-059 Cracow, Poland

**Keywords:** glassy protonic conductors, uranyl-based layered superprotonic materials, medium temperature fuel cell, mechanochemical synthesis of composites

## Abstract

The introduction of the hydrogen economy, despite its obvious technological problems, creates a need for a significant number of niche-focused solutions, such as small-sized (10–100 W) fuel cells able to run on hydrogen of lesser purity than what is considered a standard in the case of PEMFCs. One of the solutions can be derived from the fact that an increase in the operational temperature of a cell significantly decreases its susceptibility to catalyst poisoning. Electrolytes suitable for the so-called medium temperature operational range of 120–400 °C, hence developed, are neither commercialized nor standardized. Among them, phosphate silicate protonically conductive glasses were found not only to reveal interestingly high levels of operational parameters, but also, to exhibit superior chemical and electrochemical stability over their polymeric counterparts. On the other hand, their mechanical properties, including cracking fragility, still need elaboration. Initial studies of the composite phosphate silicate glasses with uranyl-based protonic conductors, presented here, proved their value both in terms of application in fuel cell systems, and in terms of understanding the mechanism governing the charge transport mechanism in these and similar systems. It was found that whereas systems containing 10–20 wt% of the crystalline additive suffer from significant instability, materials containing 45–80 wt% (with an optimum at 60%) should be examined more thoughtfully. Moreover, the uranyl hydrogen phosphate was found to surpass its arsenate counterpart as an interesting self-healing behavior of the phase structure of the derived composite was proved.

## 1. Introduction

In current times, the extensive introduction of the hydrogen economy [1] is one of the most promising answers to the issue of climate change [2] and environmental pollution caused by the use of fossil fuels. Therefore, various types of fuel cells are considered for applications, differing in both the size and the purity of the hydrogen consumed. On the other hand, the extended requirements for hydrogen purity [3] make the application of current industrial standards—PEMFCs—difficult when bioprocesses and/or on-site reforming of the natural or petrol gasses are considered as the hydrogen sources. In these cases, due to the significantly weaker CO-induced catalyst poisoning [4,5], the operation of the fuel cell at temperatures exceeding 100 °C would be a prospective technological solution [6,7]. Additional features related to such operational conditions [8] originate from the faster kinetics of the electrode reactions [9] and simplified heat management [10]. In consequence [11], the successful application of the protonic conductors operating between 150 and 400 °C is still problematic [12], independent of the fact that the high-temperature operational range of the fuel cell is widely explored. Therefore, various inorganic systems, including ceramic [13], mesoporous [14], composite [15], and, first of all, glassy protonic conductors [16], are considered potential electrolytes for this operational range of temperatures.

The particular interest in the characterization (and preceding synthesis) of the phospho-silicate (30% P_2_O_5_–70% SiO_2_) electrolyte materials originated from the need to obtain a solid membrane able to maintain the expected proton exchange rate when applied in a fuel cell design working in a middle-temperature regime (50–180 °C). Pristine membranes investigated previously by us [17,18], were obtained by means of the modification of the sol-gel process proposed originally by Nogami et al. [19,20]. Upon the hydrolysis of the appropriate orthosilicate and phosphate organic derivatives, followed by the thermally driven condensation stage occurring due to water evaporation [21,22]. Finally, the inorganic polymeric network of xerogel is formed during the annealing process of the material related to parallel occurring dehydration of the specimen and condensation of its silicate and phosphate structural units.

Similarly, for other materials of this kind, the observed protonic conductivity was strongly dependent on the content of water in the glass specimen. The values of the “dry state” conductivity of these materials span from 10^−4^ S cm^−1^ to 10^−9^ S cm^−1^, depending on the temperature and type of material undergoing the examination. In contrast to the above information, the results gathered for ‘wet’ conditions of samples reached the value of 10^−3^ S cm^−1^ with the observed variability significantly smaller than the one mentioned above. The discrepancy is easy to explain if one considers the domination of the so-called ‘vehicular mechaism’ of the proton transport in the wet state. The dependence of the mobility coefficient of these species on the hydration level correlates the conductivity with the molar ratio of water molecules to the mobile protons. On the other hand, the dehumidified glasses transport protons mostly due to the Grotthus type of behavior. It is worth noticing that the resulting materials are highly porous, and that the majority of the water molecules immobilized within the materials are located inside their internal pore space, which is characterized by average pore diameters in the range of 2–30 nm [23]. It was well determined that with the increase in water content, the value of the activation energy linearly decreases from approximately 40 kJ/mol observed at a 5% pore fill rate to 10 kJ/mol observed for a rate in a range of 90%.

On the other hand, composites with various electrically inert [24,25], as well as, protonicaly conductive additives [26,27], can form from the examined proton glassy conductor with the use of the set of preparation techniques. This includes dispersing of the solid filler in the sol, co-precipitation, as well as, solid state processes, including mechanochemistry. The latter approach is based on the idea that the application of mechanical phenomena delivers energy into the system of interest to induce the chemical reaction occurring in the solid state. In its classical meaning, this type of process is correlated with the rearrangement of the covalent bonds upon delivery of mechanical energy [28]. The transitions obtained include various inorganic functional materials, including metallic nanoparticles, magnetic materials, and metal carbides [29] or boride-based nanocomposites.

In our previous research, CsHSO_4_ was synthesized in its I, monoclinic, P21/C phase. After the ball milling process-based mixing with 70SiO_2_ 30P_2_O_5_ proton conductive glass, the final product (CsHSO_4_/phospho-silicate glass composite) was obtained. Whereas the amorphous structure of the glassy matrix remained virtually intact by the mechanochemical process; in the case of its sulfate counterpart, the phase transition between the substrate and a mechano-synthetically modified product was recognized. The original material was converted into the intermediate temperature monoclinic structure-phase II. It is worth noticing that in the bulky crystalline material, this prone to conductivity structure is appearing only in elevated temperatures above 96 °C. Contrary to this general behavior in the composite material obtained in the high-energy process, the same structure is stabilized (frozen) even at ambient temperatures.

Uranyl phosphates and arsenates of various cations (i.e., copper, calcium) belong to the most popular mineral forms in which this element is found in geological deposits. They exhibit both low solubility in water and high stability in oxidative environments. On the other hand, their protonated counterparts (HUP-(UO_2_)(HPO_4_)(H_2_O)_4_ and HUAs (UO_2_)(HAsO_4_)(H_2_O)_4_), if present in appropriate structural forms, exhibit both high concentration and high mobility of oxonium ions and, therefore, high ionic conductivity [30]. Moreover, the preparation of these compounds is relatively easy [31] and is based on diffusing solutions of UO_2_(NO_3_)_2_ and H_3_PO_4_ or H_3_AsO_4_ into large amounts of water. These structures were studied not only in terms of their charge and water transport properties [32], but as well as their dielectric properties’ variability occurring due to the phase transitions present [33] in the material and thermal properties [34]. The crystalline structure of the material responsible for the enhanced conductivity comprises layers of the polymeric form of (UO_2_PO_4_)_n_ separated by double-layered structures of water molecules, in which each fourth of them is protonated, forming the H_3_O^+^ moiety. Therefore, the room-temperature conductivity of this material reaches 4 × 10^−3^ S cm^−1^.

Similar studies were performed by Childs et al. [35], who, on the basis of the results of the NMR studies, claim that the simple mechanism of the H_2_O and H_3_O^+^ moieties cannot explain the significant enhancement of conductivity observed for the protonated compounds. Therefore, the Grotthus-type mechanism is proposed in accordance with the previous research of the same group [36]. In consequence, the activation energy of the charge transport determined for these layered materials is significantly lower (10–20 kJmol^−1^) in comparison with the one observed for the highly stiffened systems, such as ice (67 kJmol^−1^).

In another study [37], members of the same research group report anisotropy of the conductivity of the phosphate uranyl compounds. The conductivity values observed were significantly higher (4 × 10^−3^ Scm^−1^—when this value is determined upon measurements performed in a direction parallel to the faces of the disks sintered from the compound). This corresponds to the value of the activation energy being equal to 31 kJmol^−1^. On the other hand, the value determined in the perpendicular direction is 3–10 times lower, which, according to the author’s opinion, is explained by the preferred direction of orientation of the plate-like crystallites. It was also confirmed that both structures of interest revealed virtually less heat-effect (0.5 kJmol^−1^) phase transitions from tetragonal to orthorhombic symmetry, resulting in the enhancement of water molecules’ mobility at 274 (HUP) and 301 K (HUAs) [38], as well as pressure-induced structure densification [37].

Moreover, Mercier et al. describe the thermally induced transitions occurring in the HUP crystalline material. On the basis of DSC studies, as well as by their DTA counterparts [39,40], authors prove that the dehydration and decomposition of the original compound occur in two steps characterized by onset temperatures between 370 K and 435 K. It was proven that while the former effect is related to the liberation of two water molecules per molecular unit, the latter corresponds to the structural changes affecting the uranyl-phosphate subunits. The enthalpy values of these transitions were determined to be equal to 125 calg^−1^ and 175 calg^−1^, respectively. Contrary to the above information, the thermal dehydration of hydrogen uranyl arsenate, while mentioned in the literature [31,40], is described in a significantly less precise form. It was found that the first sharp and clearly observable step of the process occurs close to 360 K, while the second is significantly less pronounced in comparison to both the former, and its analog happening in HUP, which is covered with temperatures ranging from 423 to 473 K.

In terms of the research presented in this paper, even more important investigations are delivered in [5], where one can find a detailed description of the thermal properties of these materials, including their ability to lose water even at slightly elevated temperatures. Weight loss curves determined for both materials immersed in the stream of dry air prove the occurrence of water elimination even below ambient temperatures. On the other hand, the kinetics of this process are relatively slow up to a threshold temperature located at approximately 315 K, where there is an abrupt fastening of the dehydrated materials. It is, therefore, recognized that the structure of HUP can be easily modified by both interactions with water and by thermal treatment. This results in both changes in the phase transition temperature and a decrease in the values of the conductivity determined, while the XRD patterns of the altered materials remain virtually intact. Moreover, it was determined that the activation energy of the conduction process decreases significantly with the occurrence of the said structural rearrangement and reaches 0.35 eV in comparison with 0.7 eV observed in the lower temperature range. In addition to that, the highly conducting, low activation energy phase is based on the spectroscopic studies that found it to be highly disordered, with a quasi-liquid state of water molecules entrapped between the UO_2_PO_4_ layers.

Therefore, it was interesting to apply the mechano-synthetic route to composite materials based on the phosphate silicate glassy network and other crystalline protonic conductors, such as HUP and HUAs, which exhibit ‘soft’ layered structures and should be even more susceptible to changes upon delivery of the mechanical energy in comparison with the previously studied cesium-based crystalline conductors. The materials planned to be investigated here are, due to their high protonic conductivity, on the one hand, potentially applicable as components of the medium temperature fuel cell, e.g., towards the design of the structured electrode-electrolyte interface. On the other, the more basic properties of the mixtures obtained in such a manner can valuably contribute to the understanding of the deviations of the mechanism of protonic transport in the mechanically modified composite multi-component materials with more than one ionically conductive phase present.

## 2. Materials and Methods

### 2.1. Materials Synthesis

**30P_2_O_5_–70SiO_2_ raw glass** material (depicted later as RL 31) was synthesized with the use of the previously in-house developed sol-gel procedure [17] with the addition of poly(vinyl alcohol) (PVA) (M_w_ = 10^5^ g mol^−1^) (Sigma/Aldrich, St. Louis, MO, USA) acting as a burnable internal friction release agent active during the stages of sample drying and thermal annealing. The substrates used included tetraethylorthosilicate (TEOS) (Fluka, >99.0%) and trimethyl phosphate (TMP) (Acros, 99+%), both serving as the organic precursors. The reaction was held in a mixture of water (24% *v*/*v*) (Millipore, purified), ethanol (POCH, 99.8%) (75% *v*/*v*), and formamide (1% *v*/*v*) (Fluka, 99.5%) laced to pH = 3.0 with hydrochloric acid (POCH, analytical grade). The reactants were poured into the hydrolyzing solution after 2 h and were extensively stirred at 60 °C for another 24 h. The obtained gel was transferred into PTFE forms in order to evaporate the solvent. “As-received” xerogel samples were slowly dried in temperature ranging from room temperature to 35 °C for 4approximately a week and later used for the synthesis of all composite materials described herein, as well as, to prepare the G_m_ pristine material.

**Uranyl compounds** (**UO_2_**)(**HPO_4_**)(**H_2_O**)**_4_** (**HUP**) **and** (**UO_2_**)(**HAsO_4_**)(**H_2_O**)**_4_**) (**HUAs**) were synthetized according to the recipe provided by Pham-Thi et al. [41,42]. The original scheme was modified as uranyl acetate (POCh, Gliwice, Poland) and was used instead of the uranyl nitrate proposed in the literature. A 2.3 mol dm^−3^ water solution of either phosphoric (POCh, Poland) or arsenic acid (POCh, Poland) were used as precipitation agent to obtain HUP and HUAs suspensions, respectively. After sedimentation, the obtained powders were, first of all, washed with water acidified with the respective acid (0.02 mol dm^−3^), taking under consideration the solubility data delivered in [43] and carefully dried to avoid compound dehydration.

**The preparation of composites** was performed by means of the quasi-mechanochemical process. A planetary ball mill-micro mill-Pulversitte 7 Premium Line ball (Fritsch, Idar-Oberstein, Germany) was run for 3 min at 720 rpm delivering energy in the range of 600 J into the substrates (1 g in total) enclosed in a zirconium oxide grinding bowl.

### 2.2. Analytical Methods of Evaluating Samples

#### 2.2.1. DSC Experiments

Experiments were performed with Thermal Analysis Q 200 apparatus. Samples weighing from 10 to 15 mg were sealed in aluminum pans and heated from ambient temperature to 250 or 500 °C with a 10 deg min^−1^ scan rate.

#### 2.2.2. PXRD Experiments

Experiments were performed on a Bruker D8 Advance diffractometer (Bruker, Billerica, MA, USA) working with Cu K_α_ irradiation. To examine eventual irreversible thermal degradation processes in the materials two sets of samples were studied, one comprising the pristine composites while the other was prior to the thermal investigation pre-annealed in 250 °C for 24 h and later left in ambient for approximately a week to re-gain the equilibrium with humidity present in the air.

#### 2.2.3. The Electrical Impedance Spectroscopy (EIS) Measurements

Measurements were completed using an equipment set comprising EG&G Solarton Schlumberger 1255 Gain Phase/Impedance Analyzer and EG&G Solartron 1296 Dielectric Interface (Schlumberger, Paris, France). The stainless-steel measuring pan was equipped with an external heating system. The temperature range of the experiment was set from room temperature to 250 °C with 5 °C intervals and approximately 30 min equilibration time between each two subsequent measurements. In order to estimate the fragility of the pulverized-pelletized materials, studies against drying the heating cycle were interrupted for 24 h at 80 °C while the specimen investigated was annealed at this temperature without removing it from the pan. Therefore, the measurement at 80 °C was performed twice prior and after the annealing period. On the other hand, the monolithic glassy samples were investigated in a single cycle without said interruption. In both experimental variants, the impedance spectroscopy measurements were carried out without using any additional humidification systems. Samples of the pulverized composites were prepared by means of pressing performed in room temperature with 7500 kg cm^−2^ load in a 13-mm vacuum die. Reference monolithic specimens of phosphosilicate glass were cut directly from the disks of xerogel. RelaxIS software pack from RHD instruments was used for data processing and analysis. R_1_ + R_2_/Q_2_ + R_3_/Q_3_ equivalent circuit was applied to most of the spectra gathered in order to distinguish between bulk and grain boundary-related contributions to ionic conductivity. Such a separation was, on the other hand, impossible for high-temperature spectra where a simplified model (R_1_ + R_2_/Q_2_) was applied.

#### 2.2.4. FT-IR Experiments

Measurements were performed on pulverized samples using Nicolet Avatar 370DTGS FT-IR spectrometer (Thermo Electron, Waltham, MA, USA) combined with diamond ATR thermal cell TempPRO7 (Thermo Electron, USA). Data processing was performed with OMNIC^TM^ Series Software delivered by Thermo Fisher Scientific (Thermo Electron, USA). The temperature program used ranged from 40 to 200 °C. The heating rate was established at 10 deg/min, and 15 min of stabilization was applied before each spectrum acquisition. Spectra were acquired by 32 repetitions in a range between 400 and 4000 cm^−1^ and presented in interesting spectral regions. In most cases, it was between 500 and 1300 cm^−1^.

#### 2.2.5. Protonic Transference Numbers Determination

Protonic transference numbers were determined by means of the hydrogen concentration cell method in an in-house designed gas flow cell connected to EG&G PAR (EG&G PAR, Albuquerque, NM, USA) potentiostat. Platinum/silver catalytic electrodes were put on the pre-pelletized samples (6 mm diameter). The cell was running on two sets of gas mixtures containing 5/20 and 10/50 *v*/*v*% of H_2_ in an argon carrier gas. Data were processed according to the method described in our previous paper [44].

## 3. Results

### 3.1. Calorimetric Studies

**Figure 1:**
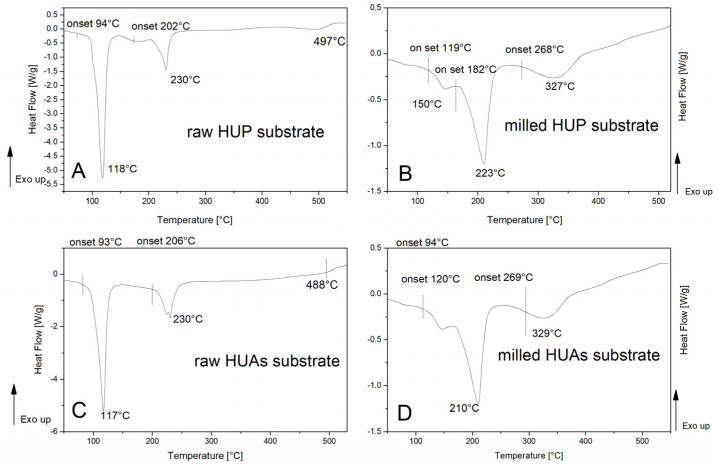
DSC traces registered for pristine materials: raw (**A**) and milled (**B**) HUP (UO_2_)(HPO_4_)(H_2_O)_4_ and raw (**C**) and milled (**D**) HUAs (UO_2_)(HAsO_4_)(H_2_O)_4_.

Results were in the first row used to verify the impact of the mechanical energy delivered, by means of milling on the properties of the pristine substrates used further in formation of the composite materials. The ‘as received’ HUP material (HUP_0_) (see Figure 1A) presents properties up to some concern, resembling those depicted in the literature [39,40], with two endothermic signals located at approximately 118 °C and 230 °C and specific enthalpies equal to 473 and 104 Jg^−1^, respectively. Pronounced deviations from both the literature delivered and the experimentally observed thermal images of the material are observed in the DSC trace of the milled material (HUP_m_) (see Figure 1B). First of all, one can notice that there is no trace of the endothermic process observed previously at approximately 118 °C, while the onset temperature of the second one is shifted towards lower temperatures by approximately 8 °C. On the other hand, the specific enthalpy of this process is increased up to 154 Jg^−1^, still not reaching the level reported in the literature. In addition to that, it must be noticed that both signals observed for the highly pulverized system are slightly wider (45 and 50 °C, respectively) than the original ones (41 and 38 °C). Moreover, the endothermic effect, originally located at 497 °C, is due to milling being not only significantly shifted towards lower temperatures (to 327 °C) but also approximately tenfold more intensive.

Similarly, in nature, deviations were observed upon the comparison of the raw (HUAs_0_) (see Figure 1C) and pulverized (HUAs_m_) (see Figure 1D) pristine HUA materials. While the low-temperature transition originally observed at 117 °C (407 Jg^−1^ and a width of 32 °C) virtually vanishes upon milling, the high-temperature one (117 Jg^−1^ and 40 °C width) that was originally split into two partially separated minima (located near 220 and 230 °C, respectively) coalesces into a significantly wider (68 °C) and stronger 219 Jg^−1^ one. Similarly, in the previous case, the characteristic temperature of this process was shifted downwards to 210 °C. Therefore, based on the results reported herein, we can suggest that the exact behavior of these materials can be dependent not only on their chemical composition but also on the ‘mechanical’ state of the particular specimen under investigation.

This observation can be easily attributed to the gradual dehydration of this material, starting from weakly bound physically adsorbed water (up to approximately 130 °C) with the subsequent desorption of strongly bound water, as well as, ongoing condensation occurring in both polyciliate and polyphosphate subnetworks of the glass. These observations are in good agreement with the results of the DTA investigations performed by us previously on the same material [17], where a broad endothermic peak starting at 100 °C and spanning up to 220 °C was observed. On the other hand, milling of the raw glass material does not induce any significant deviations in its thermogram. While both the onset and maximum temperatures of the first broad endothermic peak remain virtually unaffected, one can easily observe that the high-temperature endotherm of the processed material is clearly better separated.

In the case of the composite materials, their DSC traces can be easily grouped into two significantly different sets. First, they collect all samples between 10 and 20 wt% of the crystalline additive, independent of its chemical nature. In all these four cases, only one broad endothermic peak is observed near 100 °C. Upon closer investigation, it can be divided into two subcomponents. The first is located in a lower temperature range and can probably be attributed to the initial steps of dehydration of the glassy component, while the other contains additional contributions originating from either HUP or HUAs. On the other hand, the overall specific heats related to these transitions are significantly lower (compared with the data gathered in Table 1), than the ones characterizing the raw components of the composite. Therefore, the general nature of the changes observed upon milling in this temperature range is similar to the ones described above for the pure materials.

On the other hand, the high-temperature endothermic transition observed for both HUP and HUAs disappears entirely in the composite materials. All these observations correlate with the decrease in the thermal stability of the same set of composites observed in the PXRD experiments depicted above.

The second group of samples comprises all of these materials, which contain more than 40% of either of the crystalline components. In this case, the DSC traces of the composites can be easily derived from the ones depicting the behavior of the prospective pristine materials. In terms of the high-temperature transition, deviations from the behavior of both pristine and milled raw compounds were observed. When a HUP containing a series of composites is considered, the most significant changes occur for samples containing from 44 to 60 wt% of the crystalline phase. In this case, one can notice not only a significant decrease in both the onset and peak temperatures of the respective transition but also a dramatic drop in its specific enthalpy, from 154 Jg^−1^ observed for the milled raw material to approximately 37 Jg^−1^ (value recalculated to HUP contents) for composites (see Table 1). On the other hand, the sample characterized with the highest uranyl phosphate concentration (83 wt%) exhibits almost unchanged values of this parameter when compared to the reference one. Moreover, the observed decrease in the onset temperature occurring upon milling is 30 °C, in this case, significantly lower than the other two composites (42–54 °C), but still somehow higher than for the pristine material (20 °C). In addition to that, in the case of the former material, the peak temperature related to the transition is almost the same as for the pristine milled one (ΔT = 3 °C), while for the other two former cases, the preparation of composites affects this parameter significantly (33–41 °C). In the case of HUAs containing materials, the high-temperature transition is in all cases suppressed in terms of its effective specific enthalpy (60–100 Jg^−1^) not only if the intensity of the effect is compared with the pristine milled material (239 Jg^−1^) but even with the raw arsenouranyl hydrate (117 Jg^−1^). In addition to that, both onset and peak temperatures characterizing these systems are decreased in comparison to the raw HUAs. Still, the value of the milling-induced shift (10–20 °C) is significantly lower than the one observed for the reference sample (46 °C). Similarly, the broadening of the transition-related peak is in all three cases, observable but weaker than for the reference.

### 3.2. Powder X-ray Diffraction

Experiments were registered in order to clarify the structural issues occurring in terms of the variety and complexity of the investigated systems. Therefore, comprehensive structural studies of all individual components (see Table 1 for sample descriptors and respective compositions) were performed prior to and after a temperature treatment.

**Figure 2:**
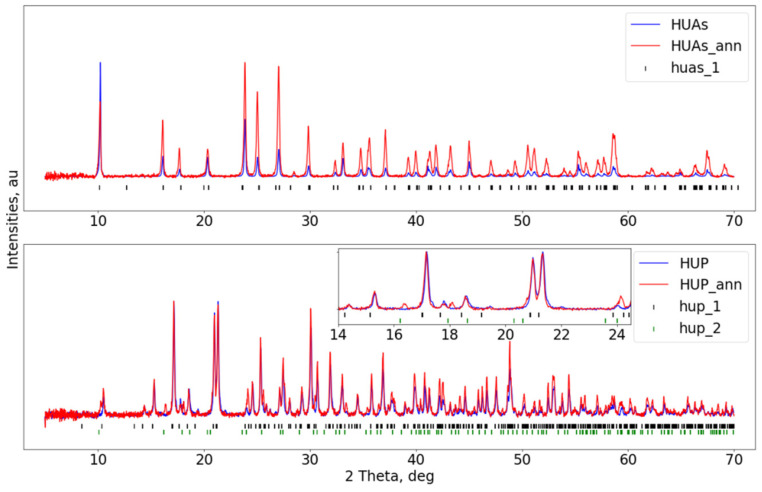
Diffractograms of raw HUAs (UO_2_)(HAsO_4_)(H_2_O)_4_ and HUP (UO_2_)(HPO_4_)(H_2_O)_4_ materials in the upper and lower sub-figures, respectively. Curves show results obtained before (**blue**) and after (**red**) annealing. Vertical bars indicate reflections’ positions of assigned crystallographic phases (see main text).

Figure 2 shows the results obtained for HUAs and HUP raw materials. Measured patterns were found to correspond to the correct phases of (UO_2_)(HAsO_4_)(H_2_O)_4_ (space group *P -1*, called here **huas_1** [45]) and (UO_2_)(HPO_4_)(H_2_O)_4_ (space group *P 4/n c c*, called here **hup_1** [39]) for HUAs and HUP, respectively. For both materials, no change in lattice constants was recorded within the experimental resolution upon annealing. However, a small fraction of the new phase appears in HUP material as a result of thermal treatment, which was found to correspond to (UO_2_)_3_(PO_4_)_2_(H_2_O)_4_ (space group *P n m a*, and further referred to as **hup_2** [46]). Post-annealing intensity variations of peaks (especially visible in HUAs samples) were attributed to the loss of occluded water. This, first of all, confirms the stability of the crystalline structure of the materials upon high-energy milling. On the other hand, both processes observed for the pristine materials upon thermal annealing comply with the information about their limited thermal stability presented in the literature cited above [31,40,42].

For both materials, no change in lattice constants was recorded within the experimental resolution upon annealing. However, a small fraction of the new phase appears in HUP material as the result of thermal treatment, which was found to correspond to (UO_2_)_3_(PO_4_)_2_(H_2_O)_4_ (space group *P n m a*, and further referred to as **hup_2** [46]). Post-annealing intensity variations of peaks (especially visible in HUAs samples) were attributed to the loss of occluded water. First of all, this confirms the stability of the crystalline structure of the materials upon high-energy milling. On the other hand, both processes observed for the pristine materials upon thermal annealing comply with the information about their limited thermal stability presented in the literature cited above [25,44,46].

**Figure 3:**
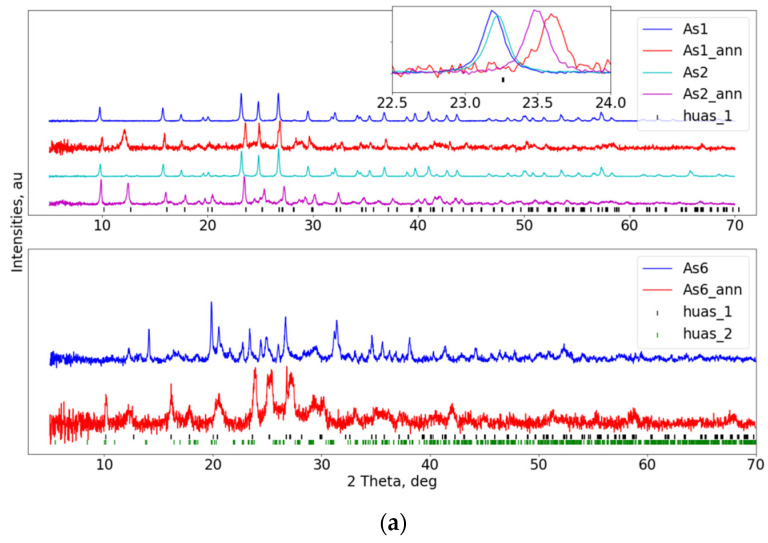

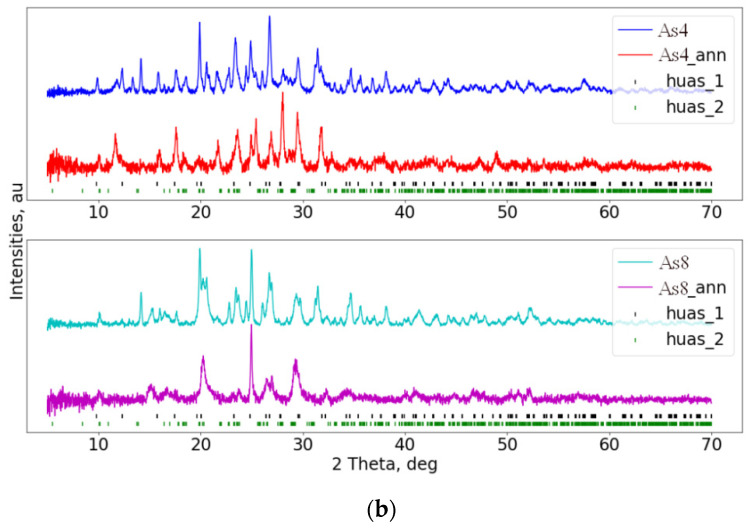
(**a**) PXRD patterns of the: As1 (90% Gm and 10% HUAs), As2 (80% Gm and 20% HUAs) and As6 (40% Gm and 60% HUAs) composites inset on an upper sub-figure shows reflection’s shift towards higher angles, indicating shrinkage of the material’s unit cell. (**b**) the As4 (54% Gm and 46% HUAs) and As8 (18% Gm and 82% HUAs) HUAs-based composites. Vertical bars show Bragg peak positions of the assigned crystallographic phases (see main text).

In the next step, composites obtained from HUAs and SiO_2_-P_2_O_5_ glass were tested by means of the analogous scheme. These were As1, As2 and As6, containing 10, 20 and 60 wt% of HUAs, respectively. Results are presented in Figure 3a. For samples As1 and As2, no structural changes have been recorded besides differences in unit cell size, which were calculated to be on the level of 0.5% of the lattice constants. In the case of sample As6, patterns are much more distorted, indicating the deterioration of an ordered crystallographic structure. Peaks originating from the new phase can be observed, interestingly, already prior to annealing. Its Bragg reflections have been attributed to the (UO_2_)(H_2_AsO_4_)_2_(H_2_O) phase (space group C 2/c, **huas_2** [47]). Although the additional phase vanishes after thermal annealing, the overall crystallinity of the sample is further degraded. A similar situation can be observed (see Figure 3b), as well, for another two HUAs containing materials. As4 and As8 composites, even prior to the thermal annealing, reveal the presence of the huas_1 and huas_2 phases. Moreover, these materials show significantly higher distortions of their crystalline structure. Thermal treatment results in a slight re-arrangement of the huas_1/huas_2 contributions, but both phases are still clearly visible in the acquired patterns.

**Figure 4:**
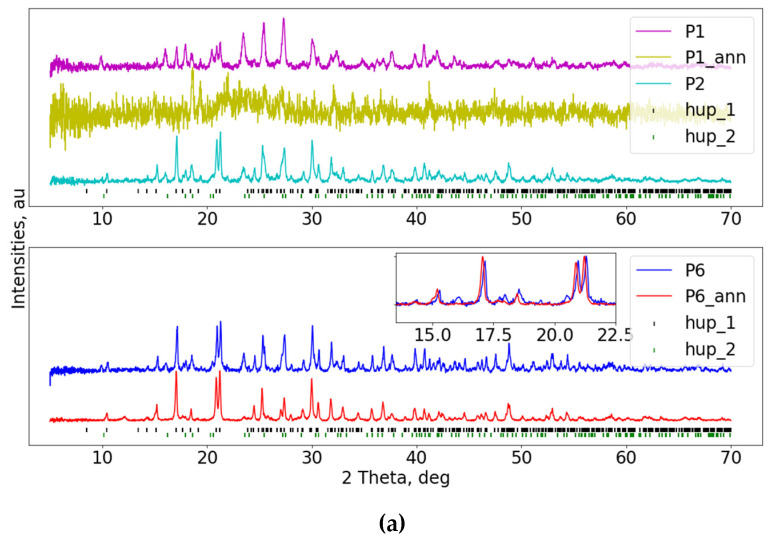

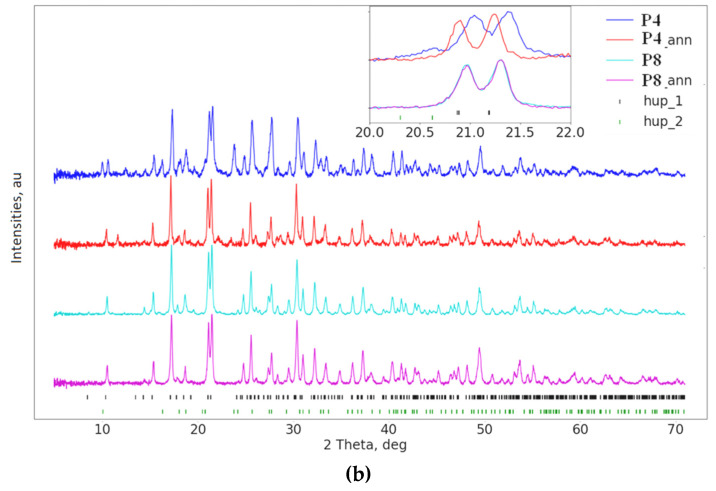
(**a**) PXRD patterns of the HUP—glass composites: P1 (90% Gm and 10% HUP), P2 (80% Gm and 20% HUP) and P6 (40% Gm and 60% HUP) inset in the lower sub-figure shows disappearance of one of the crystallographic phases, as well as slight reflection’s shift towards higher angles. Vertical bars show Bragg peak positions of the assigned crystallographic phases (see main text). (**b**) P4 (56% Gm and 44% HUP) and P8 (19% Gm and 81% HUP) inset plot show a shift of the reflections, indicating unit cell expansion in P4 sample upon annealing. Vertical bars show Bragg peak positions of the assigned crystallographic phases (see main text).

The analogous investigation was carried out for the HUP—glass mixtures, which can be seen in Figure 4a for samples P1, P2 and P6. All samples provided distorted diffractograms with both hup_1 and hup_2 phases present in patterns measured in un-processed materials. Further annealing led to the complete destruction of the long-range order and the vanishing of any traces of the initial phases (shown only for the P1 system). In contrast to HUAs-based samples, the P6 sample shows much higher quality than its two siblings. Furthermore, after annealing, a small fraction of the hup_2 phase disappears, resulting in a pure hup_1 phase, which corresponds to raw HUP. Moreover, the re-gained structure shows the shift of Bragg reflections towards lower angles, indicating expansion of the unit cell. Contrastively, for P8 material (Figure 4b), no qualitative or quantitative differences can be seen between pre- and post-annealed results. On the other hand, sample P4 shows similar behaviors to the P6 sample, where initial contamination of the hup_2 phase vanishes after annealing, followed by an increase in the unit cell.

### 3.3. Electrical Conductivity Studies

**Figure 5:**
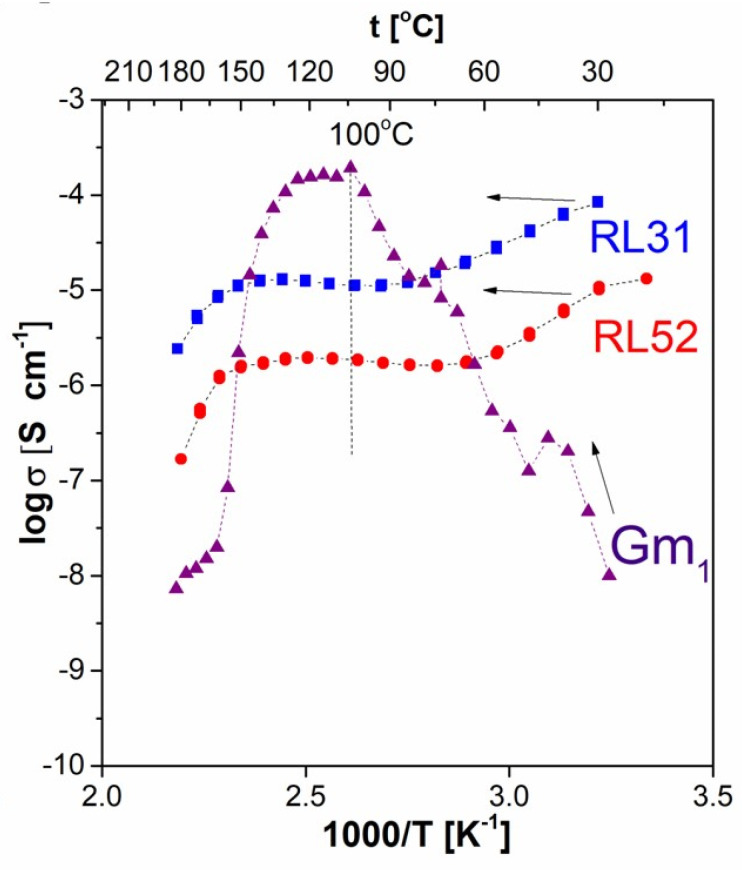
Comparison of the temperature dependencies of bulk ionic conductivity of two specimens (RL31, RL52), of monolithic PSG-30P_2_O_5_–70SiO_2_ and Gm-pelletized pulverized material of the same composition.

Studies in the first row focused on the comparison of the charge transport properties of monolithic and pulverized/pressed specimens of the pristine phosphate-silicate glass (PSG) of the same composition (30P_2_O_5_–70SiO_2_). Figure 5 presents the respective dependencies of the bulk ionic conductivity of the materials at the reciprocal temperature. RL 31 and RL 52 present here are two specimens of the monolithic PSG materials of the same composition but differ in their preparation schemes (compare [17] for details). They are presented here as a reference for the Gm obtained from the RL 31 material, the pulverized-pressed specimen obtained by means of the procedure mentioned above in the experimental section. Comparing the data, one can easily observe the discrepancies covering not only the values of conductivities but also the nature of their temperature dependence. Whereas both monolithic materials, yet differing in their specific resistivity, comply with the drying-activation-drying scheme described in detail in our previous reports [17,18], the pressed pellet of the same material does not reveal the low-temperature region of conductivity depletion. Moreover, the annealing performed at 80 °C did not lead to a significant decrease in the conductivity value. Therefore, instead of the phenomenon mentioned above, a monotonic increase in the value of the bulk ionic conductivity is observed in a temperature range spanning from ambient to approximately 100 °C (373 K), where a plateau-like maximum is observed for approximately 20–30 K. This wide maximum is followed by an abrupt decrease in conductivity related to the rapid drying of the material above this threshold point. This phenomenon is observed not only at lower temperatures than the one observed in monolithic samples but as well, leads to significantly lower conductivity values observed in the pulverized material in a wide temperature range above 140 °C (413 K). Moreover, the conductivity value observed at ambient temperature for the latter sample is, as well, significantly lower than those determined for reference, RL 31 and RL 52 materials. On the other hand, it is worth noting that in the application-focused temperature range (80–135 °C), the conductivity of the newly prepared specimen (G_m_) is one to two orders of magnitude higher when compared to RL 31 and RL 52, respectively. In addition, the activation energy of conduction calculated from the Arrhenius equation for the pulverized specimen is at least an order of magnitude higher (~1.2 eV) when compared with the ones characterized by the raw materials (0.092 and 0.093 eV, respectively).

**Figure 6:**
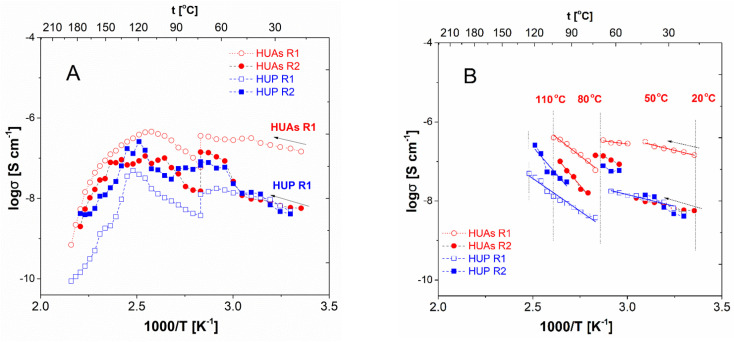
Arrhenius plot of R1 (empty symbols) and R2 (full symbols) conductivities for HUP (UO_2_)(HPO_4_)(H_2_O)_4_ (■) and HUAs (UO_2_)(HAsO_4_)(H_2_O)_4_ (●) materials; (**A**)—Arrhenius plots in a whole measured temperature range (**B**)—low and medium temperature parts of HUP and HUAs Arrhenius plots split into three independent ranges of linear dependencies.

For both the pristine crystalline materials of interest, the set of resistances gathered for pelletized milled substrates (HUP_m_ and HUAs_m_) allowed to produce the Arrhenius type dependencies of their bulk and inter-grain conductivities (Figure 6A). Unfortunately, it must be clearly stated that the pellets derived from material that underwent high-energy milling reveal a significantly (a few orders of magnitude) lower conductivity value than the one reported for the raw materials in the literature [30,48]. One should, therefore, notice that the crystalline structure of both materials remains un-tacked upon milling. This behavior was confirmed by the PXRD studies performed for both the pristine and composite materials. The significant changes were, anyhow, observed in both their respective thermal properties, as well as in the results of their FT-IR investigations. (For details on the latter, please see the data and their interpretation provided in the following section of this paper). Due to the contradictory nature of the observations gathered above, it is, therefore, hard to deliver meaningful presumptions on the nature and importance of changes occurring in the materials’ properties. Thus, to clarify the discrepancy observed, it is worth recalling the literature reports [28,29,48] claiming the susceptibility of the uranyl-based layered structures to undergo subtle structural changes. These affect their conductive properties without an observable alteration of the PXRD patterns of the respective compounds. For both materials, one can easily observe that the respective plots are clearly divided between low- and high-temperature parts. The threshold temperature where a significant drop in conductivity is observed is placed at 353 K (80 °C), the temperature at which the 24-h long annealing of the samples was performed. Independent of this fact, both presented curves depicting the thermal dependency of R_1_ derived conductivity follow, a general increase-decrease scheme related to the high-temperature dehydration of the material. This, in turn, leads to the diminishing of the protonic transport down to almost negligible values, even though at a moderate temperature range starting with the said annealing and reaching 493 K in the case of HUA-based composites and 403 K for their HUP-containing counterparts. The partially dried material exhibits a typical behavior of ionic conductors with linear changes in the conductivity with reciprocal temperature. Moreover, a similar, but even more complicated, situation is encountered in the low temperature area predominated by the transport phenomena occurring in hydrated materials. In this case, a dependency that appears monotonic at first glance, can be divided into two parts with differing slopes and activation energies. On the other hand, conductivities related to the R_2_ values in the general scope follow their R_1_-related counterparts, revealing a significantly higher level of fluctuations in the high-temperature part of the curve. The latter behavior—similar to the one revealed by the G_m_ sample—confirms to some extent the attribution of these observable inter-grain processes.

As a result of this hiaghly complicated image, a more detailed study of the activation energies, describing the thermal deviations of both conductivities, should be performed. Figure 6B presents Arrhenius-type plots of R_1_ and R_2_ related conductivity values determined for three separate temperature ranges for both compounds studied. It is worth stressing that the observed discontinuity leading to the tri-phasic form of the conductivity dependencies stays in agreement with the literature data discussed above. First of all, a structure change is reported there [38,49], leading to both the abrupt increase in the overall conductivity (the publications referred do not split the overall response of the materials studied into two frequency resolved terms) and the characteristic s-shaped dependency represented here by the response related to R_2_, while the change in the activation energy describing its changes below and above the said transition is clearly observable for both terms of interest. Moreover, whereas the absolute values of the E_a_ are here (compare Table 2 for the exact values) significantly lower than the ones reported elsewhere [49] (0.7 eV below the threshold temperature and 0.35 eV above it), the suggested tendency that the structural rearrangement leading to the liberation of the mobility of water molecules initially trapped in the inter-layer areas of the material yields in a decrease in the respective E_a_ values (excluding the values extracted from R_2_ in HUAs) is maintained. In addition to that, in all three other (complying) cases, the ratio between these two terms follows the one derived in the cited reports and is equal to approximately two. Another important observation related to this range of temperatures addresses the nature and location of the said discontinuity. Literature [38,49], reveals the temperature values characterizing the process of the water mobility enhancement as 274 K for HUP material and 301 K for its arsenate-based counterpart. Our investigation reveals this value to be significantly higher and, in both cases, settled in the range of 323–328 K. This significant shift could, therefore, suggest that the phenomenon observed is rather correlated with other changes occurring in the material upon heating—i.e., its dehydration, reported in [4] as a process of importance in temperatures exceeding 315 K. Fortunately, on the basis of premises, such as: (i) observed values of the activation energy, (ii) ratio between the said values and (iii) the characteristic s-shaped curvature of the thermal dependency of conductivity, this hypothesis can be neglected. Therefore, it is worth noticing that, in contrast to the observations mentioned above, the values of activation energy depicting the behavior of the partially dried material (>80 °C, thus, investigated after 24-h thermal annealing) are significantly higher than their respective low temperature counterparts, while the respective values of conductivity are significantly lower. This observation, yet more obviously supported by the data presented in [27,28], where a dehydration-related alteration of the conductivity mechanisms is revealed.

**Figure 7:**
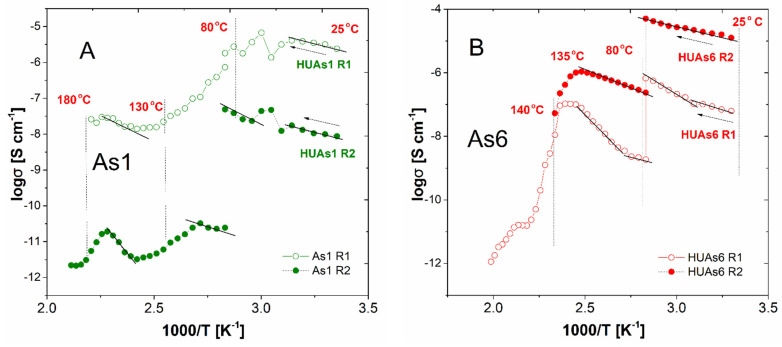
Exemplary Arrhenius type dependencies of R1 (empty symbols) and R2 (full symbols) related conductivities for: (**A**)—As1 (90% Gm + 10% HUAs)) and (**B**)—As6 (40% Gm + 60% HUAs) composite materials.

When it comes to the composite materials, the exemplary traces of their R_1-_ and R_2_-derived conductivities on the reciprocal temperature are shown in Figure 7. Prior to the detailed analysis, therefore, it is worth stressing that, similarly to the results of the PXRD and DSC studies reported above, the conductivity behaviors of these materials can be, divided into two families. On the other hand, one can easily note that despite these discrepancies, families of dependencies are, in the general view, not only similar to one another but, moreover, resemble the charge transport images obtained for the respective pristine crystalline components. Therefore, in all cases investigated, an improvement in the protonic transport is observed upon initial heating of the specimens. In the discussed temperature range (below 80 °C) one can, as well, observe a discontinuity-type phenomenon occurring at approximately 50 °C, thus, in a temperature range in which similar discrepancies are observed for the pristine HUP and HUAs materials. On the other hand, the exact nature and intensity of this deviation vary, with the composition of the materials investigated being significantly less profound in materials containing lower amounts of the uranyl-based modifiers. In addition to that (see Table 3 and Table 4 for details), the mutual relation occurring between the activation energies determined from conductivity measurements performed below and above this kink-point (but still below 80 °C where thermal annealing was performed) differs from the ones characterizing pristine materials as they are, in some cases, equal or reverse ordered with high-temperature parts exhibiting higher values than lower ones. These may prove that dehydration plays, in the case of the composite materials, an observably more pronounced role than it was observed for the pristine ones.

Important discrepancies were observed in the temperature range exceeding 80 °C, therefore, for the thermally annealed and partially dehydrated materials. For the first group of a composite comprising all these materials, which were proven to exhibit inferior stability upon thermal treatment (namely these containing 10–20 wt% of the crystalline additive, a sometimes-significant deviation from the assumed scheme of response can be observed. In an example, Figure 7A depicts the behavior of the As1 composite containing 10 wt% of HUAs. One can easily observe that in this case, a lack of the Arrhenius-type response is observed for the R_1_-derived conductivity not only above 80 °C, but slightly below this threshold. Therefore, the last five points of the low-temperature range cannot be unambiguously attributed neither to the increase nor to the decrease in conductivity upon specimen heating. Moreover, the further increase in the temperature (preceded by the thermal annealing stage) leads to a clearly linear decrease in conductivity spanning over more than two orders of magnitude. On the other hand, R_2_-related value presents at least some, but very narrow, range of temperatures related to its increase, but in both cases, the dehydration and/or degradation-related phenomena are significantly more profound, than the effect of the expected activation-related behavior. As for curiosity only, it is worth noticing that both sets of values reveal an unexpected increase in temperature ranging from 120 to 150 °C. This phenomenon of unpredictable regain of charge transport ability was observed in identical or similar form for some of the materials belonging to this group as well but, as the final values reached are in all cases very low (often lower than 10^−10^ S cm^−1^) it remains meaningless in terms of the assessment of the viability of these materials.

Contrastingly, the second group of materials (45–82 wt% of the uranyl-based additive) follows in general (compare Figure 7B, where the behavior of As6 composite is depicted) the scheme characterizing the pristine crystalline constituents of the relevant composites. Thus, it is worth to notice that the conductivity of 10^−4^ Scm^−1^ is achieved for 60 and 82 wt% containing systems in 80 °C, while at the same temperature, pellets obtained from both pristine glass (~10^−5^ Scm^−1^), as well as, both crystalline additives (<10^−6^ Scm^−1^) behave significantly inferior. On the other hand, while a similar or even higher value of maximal conductivity (1.9 × 10^−4^ Scm^−1^) can be obtained for G_m_ in 120 °C, and therefore, its thermal annealing does not lead to an observable deterioration of the charge transport ability of the material investigated, the composites which while undergoing annealing dehydrate lose their proton transport abilities. As a result, they exhibit a significant decrease in the respective σ values ranging from one to three orders of magnitude upon further increase in temperature, contrastively to G_m_. This, finally, makes them similar in behavior to HUP and HUAs specimens but, yet better conductors. In addition to that, it is worth noticing that composites based on the uranyl phosphate reveal better stability and conductivity-related performance in elevated temperatures when compared to their arsenate-based counterparts i.e., for P8 material, an almost constant value of R_1_-related ‘dry’ conductivity (~10^−5^ S cm^−1^) is maintained up to approximately 160 °C. This makes this material meaningfully superior over both its HUAs-based analog (characterized with σ = ~10^−7^ Scm^−1^), as well as, all three pristine materials (exhibiting in this temperature values approximately ~10^−8^ S cm^−1^). Moreover, the composite materials of pre-optimal (further optimization is still to be considered) composition exhibit in many cases, a lower value of the ‘dry state’ activation energy of conduction (see Table 3 and Table 4 for details). This observation, together with the, unfortunately obviously present, at least minor inconsistencies in both datasets analysed, is a strong encouragement towards the application of the described below mathematical methods of processing of the conductivity-originating data.

Therefore, in order to investigate the impact of the chemical nature of the dispersed crystalline phase on the interphase transport properties of the materials studied, an aggregated estimator was proposed. To calculate it, first of all, the ratios between the values of R_2_ (attributed here to the intergrain resistivity) and R_1_ (related hypothetically to the bulk properties of the materials studied) were calculated independently for each temperature point for each of the materials tested. It was found that, in most cases, these values lie in the range from one to a few hundred. The only observed discrepancies concerned materials (As1, P2 and to some extent As2), for which the investigations reported above (PXRD, DSC and conductivity) proved limited thermal stabilities for the composition. In these cases, the ratios were, for temperatures exceeding 80 °C (and therefore for the thermally annealed samples), either extremely high or significantly lower than one. A more detailed insight into the values obtained can serve as a probe into the existence of differences in the hindrances occurring in the intergrain charge transport between composite materials comprising components with the same (phosphate-silicate glass and uranyl hydroxyl phosphate) and various (phosphate conducting glass versus the arsenate-based counterpart of the crystalline additive) chemistries of the anionic networks corroborating in the transport of protons. If such an assumption is correct, one should observe significantly different values of the R_2_/R_1_ ratio characterizing the HUA-based family of composite materials in comparison with their HUP-based counterparts. Moreover, the values characterizing ionic transport occurring in all pristine materials should be significantly lower in comparison with the composite ones.

The averaged values of the ratios mentioned above for all the materials studied were determined separately for three various temperature ranges: (i) below 80 °C where the samples are still humidified; (ii) between 80 and 110 °C where a semi-dry state conductivity is observed but the Arrhenius type of material response is still maintained; and (iii) above 110 °C where the conductivity deterioration due to the extensive drying of the materials is revealed. These values are gathered in Table 5, showing clearly that the majority of values determined for composite materials are, on the one hand, lower than the ones characterizing the specimen obtained from the pristine phosphate-silicate glass while, on the other hand, slightly higher in comparison with those determined for pure uranyl-based compounds. Moreover, one can observe that the HUP-based materials reveal a clearly less pronounced dependence of this ratio on temperature, whereas for their HUA-based counterparts, decreased values are yielded upon material heating. In addition to that, it is worth stressing that the set of values obtained neither proves nor neglects unambiguously the assumption defined in the previous paragraph. Unfortunately, the image provided by means of such analysis is even further unclear, as the values observed for both pristine HUP and HUAs, are to some extent, lower than the ones characterizing the whole families of the respective composite materials. Moreover, while these discrepancies can be at least partially attributed to the previously described charge transport properties exhibited by the processed PSG, the comparison between the HUP and HUA-based families reveals a rather unclear image, not allowing, at this stage, to draw a meaningful conclusion. Finally, and somewhat independently (as in this particular case discrepancies are more significant, but still not clear) of the analysis performed above, it should be noticed that both samples containing 60 wt% of the uranyl compounds exhibit the highest values of the said ratios, which fits the previous observations, pointing out that this composition of materials investigated significantly stands out from all others.

Therefore, in the second row, the final aggregate was calculated according to the scheme:

(1) $$X_{H/\mathrm{As}}\;\left(T,c\right)=\left(\frac{R_{1P}/R_{2P}}{R_{1\mathrm{As}}/R_{2\mathrm{As}}}\right)$$

where:

X_H/As_—is the value of the said estimator

R_1P_ and R_2P_—are values of the respective resistivity of HUP containing sample.

R_1As_ and R_2As_—are values of the respective resistivity of HUAs containing samples

in both cases gathered in temperature T for materials containing c wt % of HUP/HUAs components.

**Figure 8:**
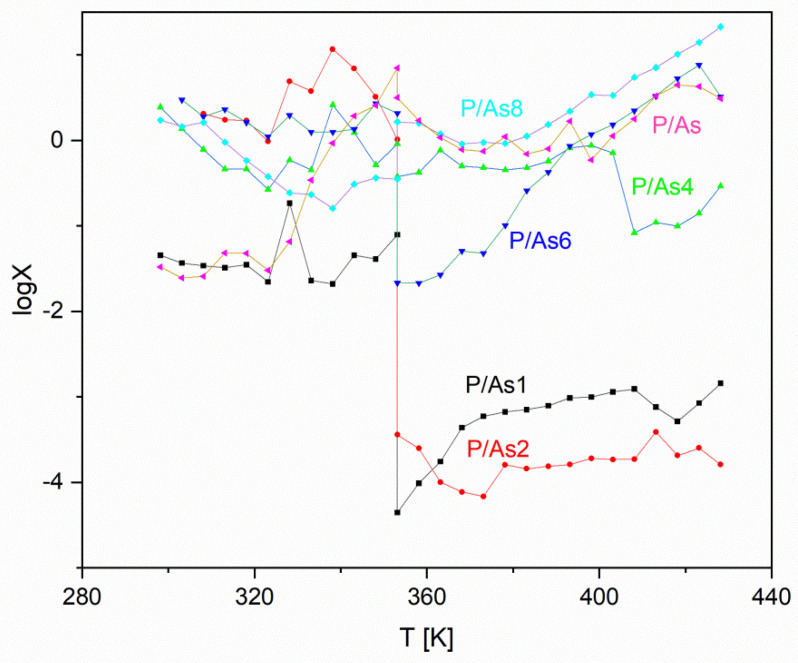
Values of the X estimator (see Equation (1) for the exact definition) are depicted as a function of temperature for all relevant pairs of the materials investigated. Composite samples: As1 (90% Gm + 10% HUAs); As2 (80% Gm + 20% HUAs); As6 (40% Gm + 60% HUAs); As8 (18% Gm + 82% HUAs) (values in w%).

The results of the calculations mentioned above are gathered in Figure 8. Despite the clearly erroneous course of the dependencies depicting materials containing 10 and 20 wt% of the uranyl compounds (compare the paragraph above and Table 5 for the explanation) and the meaningless, and therefore omitted, case of pristine glass-based material, all the values determined are in the range from 10^−2^ to 10^2^ with no clear dependence on the composition of the so-compared materials. First of all, it should be noted that, similar to the fractional ratios, the values of the final aggregate are affected by the thermal annealing performed at 80 °C as well. Moreover, the dependence determined for the pair of the pristine HUP/HUAs materials can be clearly divided into three temperature ranges virtually identical to the ones characterizing the temperature dependencies of conductivity characterizing the pristine, reference materials (compare Figure 6B for details). The value is for the lowest temperatures (below 50 °C) close to 0.03, noticing later an abrupt increase to finally stabilize close to unity for a wide range of temperatures. This proves that relations between charge transport governing phenomena differ significantly between HUP and HUAs while the wet state conductivity is concerned, occurring below the singularity point mentioned above. The markedly lower than unity value of the ratio studied proves in this case that the intergrain phenomena play a clearly less profound role in the case of the pristine phosphate-based material when compared to those revealed by its arsenate-based counterpart. On the other hand, in the case of the thermally annealed, and thus, at least partially dehydrated systems, where the ratios are almost identical, they closely resemble one another. In addition to that, it is worth stressing that in the case of the pristine materials, the change between the low- and high-temperature types of behavior is gradual and occurs between 50 and 80 °C, and therefore, prior to the thermal annealing. On the other hand, the said process does not significantly affect the values determined.

Regarding composite materials, a more detailed view of the observed phenomena should be provided. First of all, it is worth noticing that in the lowest temperature range (<50 °C), all but one of the values of the ratio determined for the composite materials are higher than the ones describing the pristine systems. To some extent, astonishingly, the values for the protruding pair (10 wt% HUx) are distinguishably closer to these, characterizing the reference compared to the others. In addition, the significantly higher set of values parameterizing the remaining systems seems to be uncorrelated with their composition. Moreover, values determined for materials containing 20 and 60% of the crystalline additive (and thus belonging to two significantly different groups of materials defined above) are those that even exceed unity. This kind of behavior, on the one hand, proves that the addition of the glassy component decreases, in this temperature range, the impact of the type of the crystalline comaterial on the importance of the intergrain phenomena. On the other hand makes the exact nature and origin of these changes unclear. An ever more complicated situation can be, as well, found in the medium temperature range (50–80 °C) where composite materials, contrastively to the pristine pair, do not reveal any significant changes in the values of the estimator analysed. This, together with the abrupt change in the behavior of the reference pair of samples, yields in a quite random situation characterized by the occurrence of values characterizing the composites both higher and lower than the quickly increasing reference. To at least partially explain this phenomenon one should consider that whereas conductivity curves for both pristine materials studied reveal change of the value of the activation energy for both (R_1_ and R_2_) components investigated the behavior of composites depends on the type of the additive applied. Most of HUP containing materials reveal here change in the values of activation energies related to both bulk and inter-grain protonic transport what made them similar to the properties of the pristine HUP material. Contrastingly, the majority of their arsenate-based counterparts exhibited a similar behavior only in terms of their R_2_ derived properties what distinguish them from the pristine HUAs sample. Finally, due to the parallel presence of both of these phenomena, the overall change in the mutual relations of values characterizing composites and reference system observed at temperatures between 50 °C and 80 °C cannot be unambiguously attributed to the behavior of neither composites nor the reference materials.

Therefore, one can easily notice, as well, that contrastively to the pristine materials described above almost all dependencies reveal more or less significant and abrupt change occurring upon annealing held in 80 °C. These changes are, obviously, related to the uneven thermal dehydration of the samples compared. The observed discontinuity is extremely pronounced (reaching approximately four orders of magnitude) for both compositions (10 and 20 wt./%) for which a stability deprivation issue was recognized during previous investigations. On the other hand, the behavior of the 60 wt.% composition exhibited upon thermal annealing is significantly different from its 45 and 82 wt.% counterparts. While in the former case and approximately two orders of magnitude shift is revealed the changes occurring in the behavior of two later sets of samples do not exceed one order of magnitude. The situation clarifies upon the thermal annealing of the materials, and therefore, their progressing dehydration. In these conditions (80–120 °C) the datasets for 45 and 82 wt.% pairs virtually follows the behavior of the pristine materials for which values close to unity are observed in whole range of considered temperatures. Contrastively all other pairs of the composite materials reveal at least somehow lower values of the investigated estimator in comparison to the reference pair of systems. Of course, the outstanding and temperature-independent differences observed for the 10 and 20 wt.% pairs proof only their weird behavior related to the unstable nature of these materials.

On the other hand, the behavior of the 60 wt.% systems for which, notwithstanding its stability, a significantly lower value is observed up to approximately 120 °C should be discussed separately. Here the deviations of properties of the ionic transport occurring in medium temperature range can lead to the assumption that for this particular (and assessed as optimal) composition, differences between HUP and HUAs-based composite are meaningfully pronounced up to significantly higher values of temperature than it happened for other stable composites investigated. Even though, upon further heating, the said difference vanishes as well. Finally, the coalescence of all the meaningful values observed occurs at approximately 120–130 °C as regarding in the same degree, both composites and the pristine reference pairs being observed. These observations make it doubtful that the initial assumption of the inter-grain proton transport properties being dependant on the chemical composition of the mixed materials. If the difference in the properties of the phosphate-phosphate and phosphate-arsenate anionic sub-lattice interfaces had affected the properties of the grain boundaries between the phosphate silicate glass and crystalline additive in a significant manner, it would have led to the situation opposite to the one observed. In such a case, the composites should reveal a significantly higher value of the discussed estimator compared to the reference pair composed of pristine materials. These observations depict the situation independently of the described in the literature i.e., in [2,4,7,27,28] changes in the dominating conductivity mechanism exhibited by both HUP/HUAs and PSG materials upon drying. Finally, it is worth noticing that the possible, but yet only very provisional, explanation of the said behavior can be derived from the possible chemical interactions of the phosphate sub-lattice of the glassy matrix with anionic moieties present in both crystalline materials. This is indicated by both the PXRD experiments described above, as well as, the results of the preliminary spectroscopic investigations of the selected systems of interest presented below.

### 3.4. Fourier Transformed Infrared Spectroscopy-FTIR

Results of the investigations of both uranyls HUP and HUAs mechanochemically obtained composites with phosphate silicate glasses (SiO_2_-P_2_O_5_), as well as, for the respective pristine materials, were gathered during in situ temperature experiments ranging from approximately 52 °C to maximally 220 °C.

The electrical impedance spectroscopy (EIS) of the materials presented in a previous chapter indicated significant conductivity changes. Part of them is typical for superionic conductors, but conductivity drops were also identified in the temperature EIS analysis, suggesting the structural reconfiguration of the composites with temperature. The mechanisms standing behind these changes are still unclear, especially in the case of the class of mechanochemically obtained phospho- and arseno- uranyl glassy composites.

Therefore, the analysis was performed in order to clarify the aspects of a molecular structure in selected temperature ranges as well as to indicate the character of these phase transitions.

**Figure 9:**
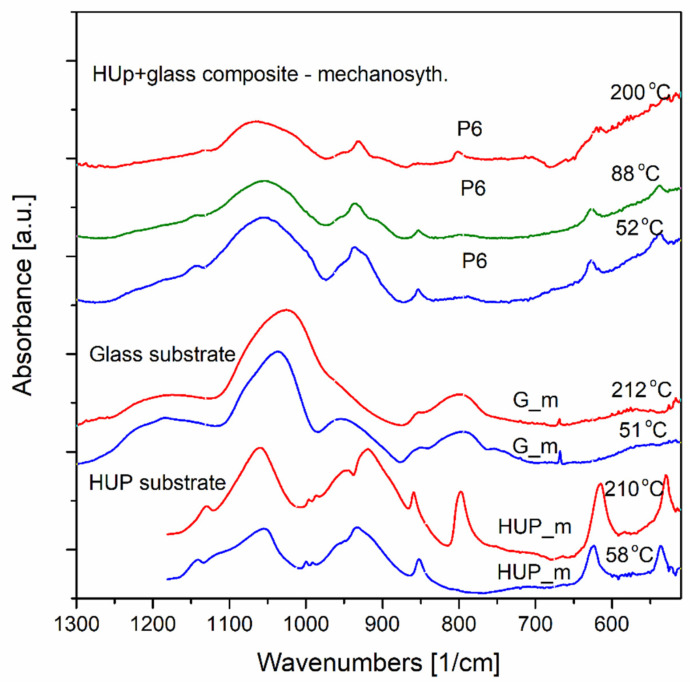
FTIR-ATR spectra of P6 composite samples (40% Gm + 60% HUP) and their substrates (HUP) (UO_2_)(HPO_4_)(H_2_O)_4_ and Gm-milled 30P_2_O_5_–70SiO_2_ glass. Spectra acquired at elevated temperatures. The spectra were normed and shifted for clarity.

**Figure 10:**
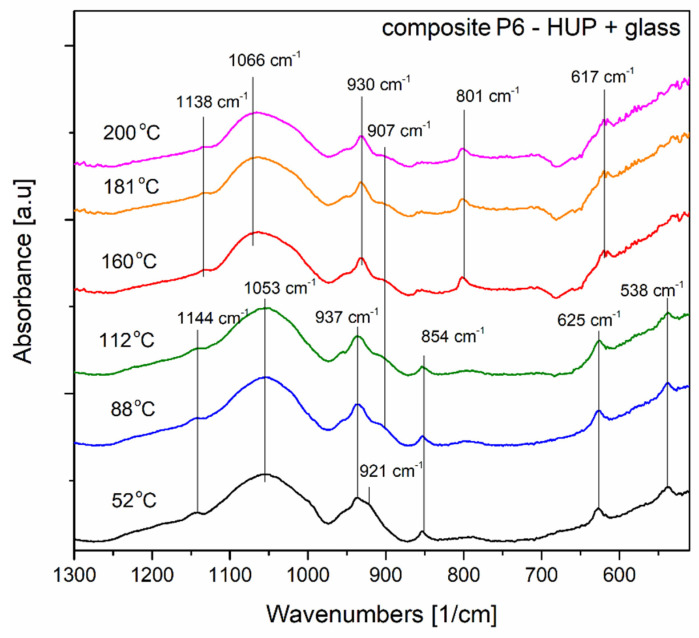
FTIR-ATR spectra of P6 composite samples (40% Gm + 60% HUP) acquired at elevated temperatures. The prominent maxima positions were marked, and the spectra were normed and shifted for clarity.

**Figure 11:**
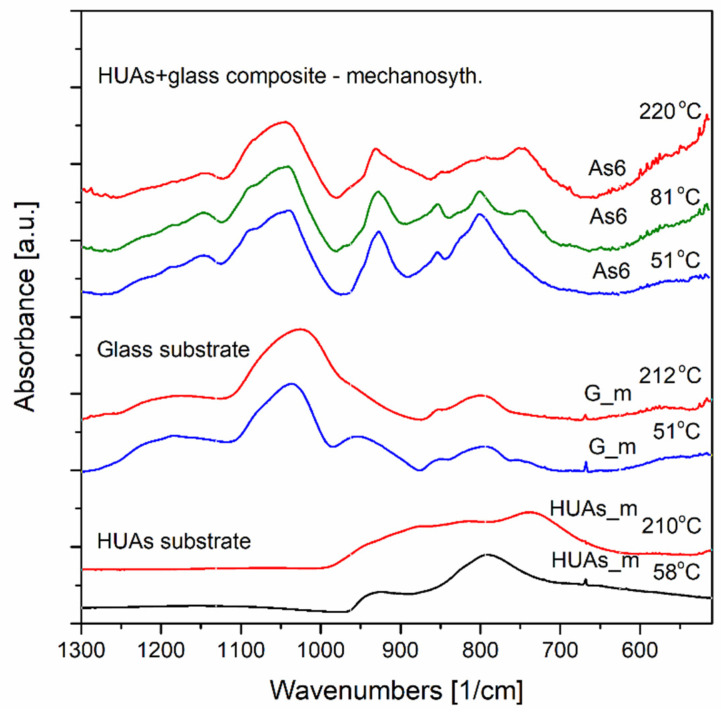
FTIR-ATR spectra of As6 composite samples (40% Gm + 60% HUAs) and their substrates: (HUAs) (UO_2_)(HAsO_4_)(H_2_O)_4_) and Gm-milled 30P_2_O_5–_70SiO_2_ glass. Spectra were acquired at elevated temperatures, and the spectra were normed and shifted for clarity.

Therefore, as it was mentioned above, the analysis was performed for substrate materials as well as for the selected composites called P6 and As6 (see Table 1 for exact compositors of these materials). The composites contain 60 wt.% of HUP and 40 wt.% of SiO_2_-P_2_O_5_ glass G_0 for P6 sample and 60 wt.% of HUAs and 40 wt.% of SiO_2_-P_2_O_5_ glasses G_0 (Figure 9, Figure 10 and Figure 11).

The results for HUP reference and P6 composite materials have been analysed mostly based on the data gathered in the paper of Kobets and Umreiko [50]. They presented the molecular spectroscopy investigations of various uranyl compounds and their phase transitions according to dehydration processes. The bands typical for two uranyl phases have been identified in the composite material P6. In temperature range up to 112 °C, mainly vibrations of UO_2_HPO_4_∙4H_2_O are present together with the phpospho- silicate glass component bands (asymmetrical stretching vibrations of Si-O bonds and asymmetrical stretching vibrations of (PO_4_)^3−^ tetrahedron).

Above this temperature, the dehydrated uranyl (UO_2_)_3_ (H_2_PO_4_)_2_ H_2_O phase is more pronounced, and it is also present in a companion of phospho-silicate glass species vibrations. 

Figure 9 presents the substrate materials and the composites of HUP. The spectra were collected in situ in temperatures near 50, 90 and 200 °C. As illustrated in Figure 9, the HUP substrate transformations differ from those observed for HUP- glass composite while both specimens undergo the same temperature regimes. In the composite samples, as obtained by a high-energy ultra-fast ball milling chemosynthesis, the structural changes occurred, promoting different thermal appearances compared to its substrates and other phase transitions of the composites. 

The HUP substrate maintains the low wavenumbers bands shape. From 58 °C up to 210 °C, the maxima near 538 cm^−1^ and 625 cm^−1^ are present in a whole temperature range. Contrary, for the composite P6, these maxima are no longer current at 200 °C.

For the higher wavenumbers ranging from 750 to 900 cm^−1^ in the HUP samples, we may see only the 854 cm^−1^ band in this range. The band shifts to the higher frequencies (to 860 cm^−1^) with the temperature increase, and the new band near 800 cm^−1^ appears at 210 °C, indicating the phase transition of the HUP sample. Similarly, for the same frequencies, this new maximum is also visible at 200 °C in a P6 composite.

Figure 10 presents the thermal evolution of the P6 composite spectra taken at six temperatures near the transitions indicated in thermal analysis (DSC). The exact band positions have been displayed in the figure to facilitate the description of the spectra’ evolution.

At the temperatures between 52 °C and 112 °C, there is no evident difference in the spectra appearance except the disappearance of the 921 cm^−1^ band attributed to ν_3_ (UO_2_)^2+^ typical for UO_2_HPO_4_∙4H_2_O according to Kobets and Umreiko bands assignment [50] same authors also assigns bands with maxima near 538 cm^−1^ and 625 cm^−1^ to ν_3_ bending vibrations of (HPO_4_)^2−^ groups of the same structure. The band near 937 cm^−1^ could also be attributed to ν_3_ (UO_2_) ^2+^ but rather in the (UO_2_)_3_ (H_2_PO_4_)_2_ H_2_O structure. This structure is even better pronounced in the temperature range above 112 °C due to the continuation of the combination of the dehydration and chemical conversion processes. In Figure 11, more bands of (UO_2_)_3_ (H_2_PO_4_)_2_ H_2_O are visible for spectra recorded at temperatures 160 °C, 180 °C, and 200 °C. The bands better pronounced in this temperature range are 618 cm^−1^, the ν_1_ vibration of (PO_4_)^3^, 801 cm^−1^, which may be attributed to ν_1_ (UO_2_)^2+^ and the 930 cm^−1^ a vibration of ν_3_ (UO_2_)^2+^ band. The band with a maximum near 1066 cm^−1^ could be assigned to HPO_2_^−^ in dehydrated (UO_2_)_3_ (H_2_PO_4_)_2_ H_2_O enveloped with Si-O and (PO_4_)^3−^ asymmetric stretching vibrations of the glassy component of the composite. This observation can be related to the hypothetic reaction of the uranyl phosphate compound with the phosphate-based acidic moieties originating from the glassy matrix being somehow confirmed by the self-healing properties of the P6 material observed within the thermal annealing-based PXRD investigations reported in Section 3.2.

On the other hand, Figure 11 presents the results of the preliminary spectroscopic investigations of both substrates and as 60 composite. The results given similarly as in the former case originate from in situ temperature performed in the same temperature range and grid. First of all, it is worth noticing that, in this case, the shapes and the positions of the comparable absorption bands differ between the composite and the substrates. This may suggest that new molecular configurations have been formed chemosynthetically.

**Figure 12:**
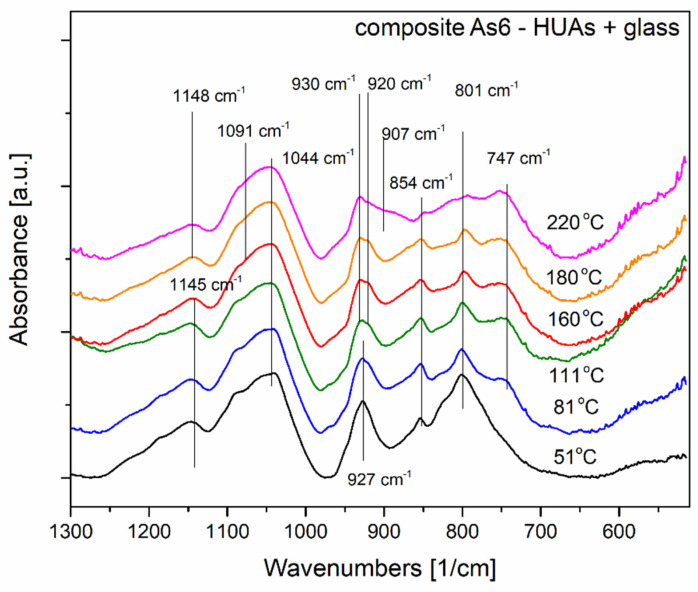
FTIR-ATR spectra of As6 composite samples (40% Gm + 60% HUAs) at elevated temperatures. The prominent maxima positions were marked, and the spectra were normed and shifted for clarity.

Moreover, it can be concluded (please refer to both Figure 11 and Figure 12 for details) that the material undergoes some thermal changes, which are consistent with the electrical impedance changes and the DSC results. The transitions we may observe are in three main stages. Up to 51 °C. Between 81 °C and 180 °C and above 180 °C (the spectrum of a significant difference was registered at a temperature of 220 °C).

First, thermally introduced spectrum changes are visible by the band’s appearance with a maximum of approximately 747 cm^−1^ at 81 °C. The band stays present up to the temperature of 220 °C. In a uranium arsenate substrate, it is attributed to (AsO_4_)^3−^. The second and third structural changes are manifested at a spectral region of 890–980 cm^−1^. The sharp band with a maximum near 927 cm^−1^ is well visible in a composite sample at 51 °C temperature. In a substrate material near this position (at 930 cm^−1^), UO_2_^2+^ vibrations are observed. At temperatures starting from 111 °C, two bands are visible instead, the 920 cm^−1^ and 930 cm^−1^. This phenomenon may also be connected to the changes in the molecular surrounding of UO_2_^2+^ ion. Finally, at 220 °C, the band shape of the region undergoes further changes and a plateau below 930 cm^−1^ became visible, possibly due to the appearance of a new maximum near 907 cm^−1^ and, most possibly, again, according to a change in molecular order of the uranium surrounding. It seems that a more comprehensive evaluation of structural changes of the material is needed since there are not many molecular structure studies on the pristine HUAs material and its composite apart from its phosphate analogues which we also have been using as a reference.

### 3.5. Protonic Transference Numbers

**Figure 13:**
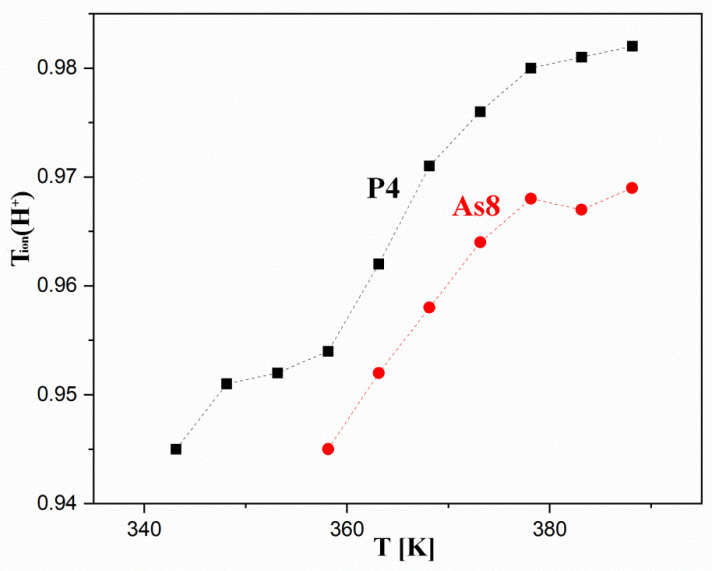
Values of protonic transference numbers determined for P4-(56% Gm + 44% HUP) in w% and As8-(18% Gm + 82% HUAs) composites as a function of the materials temperature.

The investigations on the proton transport mechanism were carried out for two composite samples P4 (44 wt% HUP) and As8 (82 wt% HUAs). First, the investigation showed a dominant proton conductivity (H^+^) in the investigated samples. The estimated transfer number (H^+^) in the temperature range of 70–115 °C varied between 0.94–0.98. A slight increase in the proton transfer number is observed with increasing temperature (Figure 13). Above 100 °C, a flattening of this tendency is observed, which, together with other experimental problems in measurements at higher temperatures, suggests that the observed increase is related to the dehydration of the material

Consequently, condensation occurring in phosphate (and/or arsenate) based anionic sub-lattices, reducing the mobility of the anionic species and, therefore, promotes the impact of the cationic transport in relation of the overall conductivity of the material.

In addition, slightly lower values were estimated for the As8 sample. Unfortunately, no clear explanation for the significance and nature of this discrepancy can be derived at this stage. However, it can be assumed that it is to some extent related to the overall poorer stability-related performance of the HUAs containing composites compared to their HUP -containing counterparts. Further electrochemical studies are needed to explain these phenomena.

## 4. Conclusions

First of all, it is worth noticing that all the results presented clearly prove the assumption that the mechano-chemical treatment of the materials studied affects their properties even when pristine components are considered. In the case of pure phosphate silicate glass, an order of magnitude higher values of the conductivity was determined for the milled-pressed material in the medium temperature range (100–130 °C), when compared to the monolithic reference specimen of the amorphous material characterized with the same chemical composition and preparation procedure. On the other hand, the treatment involved leads to an order of magnitude increase in the respective activation energy which stays in contradiction to the general rule of thumb describing the charge transport properties of ionic conductors. As a result, the room temperature conductivity of the powdered material is significantly lower when compared with the one exhibited by its initial form. Moreover, significant but differing in nature deviations are observed for both pristine crystalline materials upon milling. They reveal opposite changes in their properties, including decrease in their conductivity, as well as, an approximately twofold decrease in the respective activation energies. In addition to that, an approximately 30 °C increase in the temperatures of the structural transition specific to them is observed. This proves that the power milling process, when applied, leads to significant changes in the microstructure of the material—probably due to the high fragility of the layered structure characterizing both compounds considered.

A similar conclusion can be, as well as, derived for all the composites studied. In this case the nature of the observed changes strongly depends on the composition of the materials investigated. While low (10 to 20 wt%) contents of both crystalline additives result in systems of limited stability and, therefore, inferior properties. This observation was confirmed by both PXRD and DSC studies, where a significant deterioration of the structural properties of these materials was observed upon their thermal annealing, as well as, by their meaningfully different charge transport properties. It is worth stressing that the properties of these composites are, unfortunately, at least in some temperature ranges, worse than the respective behavior of their respective pristine building blocks. Fortunately, an opposite situation can be observed if higher (44 to 82 wt%) amounts of the uranyl-based compounds are considered. Within this set of the compositions investigated the 60 and 82 wt% ones were found to be optimal due to their superior thermal stability confirmed by the PXRD and DSC studies. Unfortunately, due to the limited amount of the glassy material acting as a binder, the samples of latter composition, while pelletized, exhibit significantly inferior mechanical properties, which at the moment focuses the interest on the former one. This of course, does not neglect the possibility of further improvement of the properties of materials upon the densification of the composition grid around this point, as well as, the modification of the preparation procedure. Moreover, it was found that the materials of the same composition differ significantly, when arsenate and phosphate-based compositions are compared with clearly better operational properties revealed by the HUP-based ones. Despite of the improved thermal stability of the phosphate-based systems, this results not only in slightly higher values of the protonic transference numbers registered, but as well, in the self-healing properties of the P6 material revealed in its PXRD and spectroscopic investigations.

On the other hand, it must be stressed that all systems investigated seem to be extremely sensitive to even minor deviations in the experimental procedures undertaken, and therefore, all future investigations (spectroscopic, conductivity in the humidified conditions, concentration cell performance) and preparatory (optimization of the mechano-chemical procedure involved) works must be planned and performed with extreme care.

## Figures and Tables

**Table 1:** Stechiometry and calorimetric data gathered and determined for the selected materials studied.

Sample	% Glass	% HUP	% HUAs	Tl _ons_ [°C]	Tl _max_ [°C]	Th _ons_ [°C]	Th _max_ [°C]	Q_l_ [J/g]	Q_h_ [J/g]
G_0_	100	0	0	75	123.4	193	210	225	-
G_m_	100	0	0	50	123	197	200	-	-
HUP_0_	0	100	0	104.4	118.3	211.85	230.5	476.8	10.7
HUP_m_	0	100	0	128	149	191.85	222.6	17.7	153.7
P1	90	10	0	62	96	-	-	69.7	-
P2	80	20	0	96.9	118.7	-	-	51.13	-
P4	56	44	0	92.6	116	169.85	188.9	128.2	16.8
P6	40	60	0	94.2	111.5	157.85	181	87.1	21.7
P8	19	81	0	75	122	176.85	219	41	126
HUAs_0_	0	0	100	103	117	219.85	230.3	407.2	116.6
HUAs_m_	0	0	100	120	148	173.35	210.3	20	219.3
As1	90	0	10	120	130	-	-	132	-
As2	80	0	20	108	122.2	-	-	144.2	-
As4	54	0	46	78.7	105	197.65	228.9	127.2	36.8
As6	40	0	60	94	113.9	210.85	231	160.3	35.8
As8	18	0	82	88.2	116.9	207.45	219.6	103.4	82.2

Symbols: -Tl _ons_—onset temperature of low-temperature transformation; -Tl _max_—maximum temperature of low-temperature transformation; -Th _ons_—onset temperature of high-temperature transformation; -Th _max_—maximum temperature of high-temperature transformation; -Q_l_—specific heat of transformation ‘low’; -Q_h_—specific heat of conversion ‘high’.

**Table 2:** Values of activation energies determined for HUP and HUAs milled-pelletized materials on the basis of three separated temperature ranges.

Sample	Ea Based on:	(20–50 °C) Ea [eV]	(50–80 °C) Ea [eV]	(80~110 °C) Ea [eV]
HUP	R1 R2	0.45 0.48	0.20 0.26	0.52 0.82
HUAs	R1 R2	0.23 0.21	0.13 0.37	0.74 0.66

**Table 3:** Immittance investigations derived charge transport parameters for the systems studied for R_1_ resonator.

			R_1_					
	25–80 [°C]		80–135 [°C]		104–230 [°C]		25–235 [°C]	
Sample	E_a_ Low	Log σ_0_	E_a_ Average	Log σ_0_	E_a_ High	Log σ_0_	E_a_ All	Log σ_0_
	[eV]	[S/cm]	[eV]	[S/cm]	[eV]	[S/cm]	[eV]	[S/cm]
Gm	1.20	12.12	1.19	12.12	1.19	12.12	1.19	12.12
As1	0.32	0.18	0.32	0.18	0.86	0.18	0.32	0.18
As2	0.30	−1.56	0.30	−1,56	0.19	−1.56	0.30	−1.56
As4	0.63	4.36	1.18	11.6	0.79	0.72	0.43	0.72
As6 As8	0.23 0.20	−0.99 −2.05	0.23 0.20	−0.99 −2.05	0.38 0.22	−0.99 −2.05	0.23 0.20	−0.99 −2.05
HUAs	0.23	−2.88	0.12	−4.59	0.73	−4.46	0.13	−4.46
P1 P2	0.34 0.20	0.39 2.74	0.24 0.26	−0.57 1.46	0.26 0.61	2.05 2.12	0.43 0.23	2.05 2.12
P4	0.15	−4.24	0.10	−4.76	0.43	−4.06	0.16	−4.06
P6 P8 HUP	0.28 0.62 0.45	−0.17 2.50 −0.84	0.28 0.56 0.20	−0.17 1.76 −4.81	1.05 0.26 0.52	−0.17 2.87 1.59	0.28 0.63 0.60	−0.17 2.87 1.59

**Table 4:** Immittance investigations derived charge transport parameters for the systems studied for R_2_ resonator.

			R_2_					
	20–80 [°C]		80–125 [°C]		105–230 [°C]		25–240 [°C]	
Sample	E_a_ Low	Log σ_0_	E_a_ Average	Log σ_0_	E_a_ High	Log σ_0_	E_a_ All	Log σ_0_
	[eV]	[S/cm]	[eV]	[S/cm]	[eV]	[S/cm]	[eV]	[S/cm]
Gm	1.06	8.22	1.06	8.22	1.06	8.22	1.06	8.22
As1	0.21	−3.53	0.39	−0.54	0.73	2.12	0.37	−1.04
As2	0.66	2.57	1.22	11.28	0.05	−6.01	1.04	8.55
As4	0.53	1.34	1.63	17.95	0.98	4.31	0.79	5.56
As6 As8	0.23 0.28	−3.29 −3.29	0.57 0.28	2.07 −3.29	1.09 0.20	5.02 −7.78	0.46 0.28	0.31 −3.29
HUAs	0.21	−4.66	0.36	−1.57	0.66	1.63	0.62	1.90
P1 P2	0.87 0.37	7.17 −1.79	0.18 0.69	−2.80 3.33	0.59 0.70	2.27 0.92	0.83 0.66	6.55 2.85
P4	0.16	−4.97	0.61	2.44	0.56	0.19	0.57	1.72
P6 P8 HUP	0.55 0.61 0.47	1.56 2.51 −0.42	0.55 0.56 0.26	1.57 1.76 −3.33	0.38 0.27 0.87	−2.01 −2.62 4.22	0.55 0.63 0.19	1.57 2.87 −5.03

**Table 5:** Average values of the R_1_/R_2_ ratio in various temperature ranges determined for all the systems studied.

		wt % of HUx Compound						
**Series**	**T [^o^C]**	**0**	**10**	**20**	**44**	60	81	100
HUP-PSG	samples	G_m_	P1	P2	P4	P6	P8	HUP
	t < 80	1.5 ∙ 10^2^	5.9 ∙ 10^1^	5.7 ∙ 10^1^	7.0 ∙ 10^0^	2.8 ∙ 10^2^	6.6 ∙ 10^0^	3.6 ∙ 10^0^
	80 < t< 110	1.7 ∙ 10^2^	7.8 ∙ 10^0^	5.3 ∙ 10^−4^	8.6 ∙ 10^0^	6.3 ∙ 10^0^	1.9 ∙ 10^0^	6.5 ∙ 10^0^
	t120	5.4 ∙ 10^1^	2.8 ∙ 10^1^	1.6 ∙ 10^−4^	4.4 ∙ 10^0^	2.1 ∙ 10^1^	3.3 ∙ 10^0^	6.4 ∙ 10^0^
HUAs-PSG	samples	G_m_	As1	As2	As4	As6	As8	HUAs
	t < 80 80 < t< 110	1.5 ∙ 10^2^ 1.7 ∙ 10^2^	1.9 ∙ 10^2^ 1.0 ∙ 10^4^	2.1 ∙ 10^1^ 3.3 ∙ 10^0^	1.1 ∙ 10^1^ 1.8 ∙ 10^1^	1.5 ∙ 10^2^ 1.1 ∙ 10^2^	8.5 ∙ 10^1^ 6.7 ∙ 10^0^	1.8 ∙ 10^1^ 5.0 ∙ 10^0^
	t > 120	5.4 ∙ 10^1^	3.6 ∙ 10^3^	8.5 ∙ 10^−1^	1.3 ∙ 10^1^	1.5 ∙ 10^1^	5.0 ∙ 10^0^	3.5 ∙ 10^0^

## Data Availability

Data available on request due to restrictions eg privacy or ethical. The data presented in this study are available on request from the corresponding author.
